# Defining the relationship between *Plasmodium falciparum *parasite rate and clinical disease: statistical models for disease burden estimation

**DOI:** 10.1186/1475-2875-8-186

**Published:** 2009-08-05

**Authors:** Anand P Patil, Emelda A Okiro, Peter W Gething, Carlos A Guerra, Surya K Sharma, Robert W Snow, Simon I Hay

**Affiliations:** 1 Spatial Ecology and Epidemiology Group, Tinbergen Building, Department of Zoology, University of Oxford, South Parks Road, Oxford OX1 3PS, UK; 2 Malaria Public Health and Epidemiology Group, Centre for Geographic Medicine, KEMRI – Univ. Oxford – Wellcome Trust Collaborative Programme, Kenyatta National Hospital Grounds, P.O. Box 43640-00100 Nairobi, Kenya; 3 National Institute of Malaria Research (Indian Council of Medical Research), Field station, Sector-5, Rourkela-769002, Orissa, India; 4 Centre for Tropical Medicine, Nuffield Department of Clinical Medicine, University of Oxford, CCVTM, Oxford, OX3 9DS, UK

## Abstract

**Background:**

Clinical malaria has proven an elusive burden to enumerate. Many cases go undetected by routine disease recording systems. Epidemiologists have, therefore, frequently defaulted to actively measuring malaria in population cohorts through time. Measuring the clinical incidence of malaria longitudinally is labour-intensive and impossible to undertake universally. There is a need, therefore, to define a relationship between clinical incidence and the easier and more commonly measured index of infection prevalence: the "parasite rate". This relationship can help provide an informed basis to define malaria burdens in areas where health statistics are inadequate.

**Methods:**

Formal literature searches were conducted for *Plasmodium falciparum *malaria incidence surveys undertaken prospectively through active case detection at least every 14 days. The data were abstracted, standardized and geo-referenced. Incidence surveys were time-space matched with modelled estimates of infection prevalence derived from a larger database of parasite prevalence surveys and modelling procedures developed for a global malaria endemicity map. Several potential relationships between clinical incidence and infection prevalence were then specified in a non-parametric Gaussian process model with minimal, biologically informed, prior constraints. Bayesian inference was then used to choose between the candidate models.

**Results:**

The suggested relationships with credible intervals are shown for the Africa and a combined America and Central and South East Asia regions. In both regions clinical incidence increased slowly and smoothly as a function of infection prevalence. In Africa, when infection prevalence exceeded 40%, clinical incidence reached a plateau of 500 cases per thousand of the population *per annum*. In the combined America and Central and South East Asia regions, this plateau was reached at 250 cases per thousand of the population *per annum*. A temporal volatility model was also incorporated to facilitate a closer description of the variance in the observed data.

**Conclusion:**

It was possible to model a relationship between clinical incidence and *P. falciparum *infection prevalence but the best-fit models were very noisy reflecting the large variance within the observed opportunistic data sample. This continuous quantification allows for estimates of the clinical burden of *P. falciparum *of known confidence from wherever an estimate of *P. falciparum *prevalence is available.

## Background

**Figure 1:**
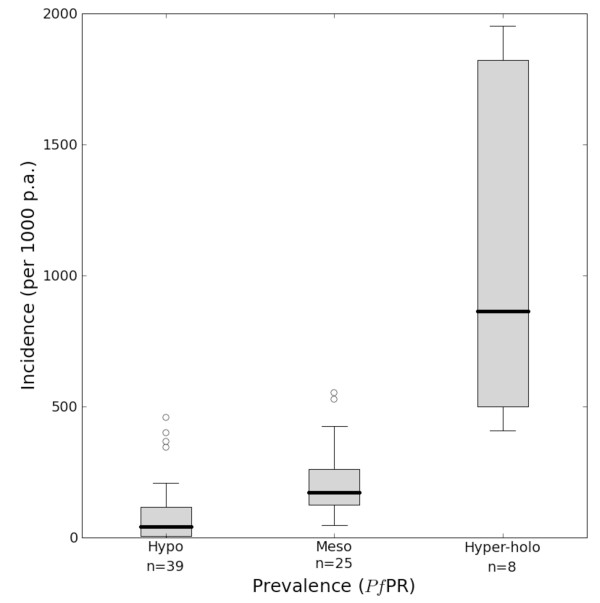
**Annual clinical incidence of *P. falciparum *per 1,000 population in hypoendemic, mesoendemic and combined hyperendemic and holoendemic prevalence conditions **[10]. The box indicates the inter-quartile range (25% and 75%) and the thick line within the box represents the median. The whiskers represent the 2.5% and 97.5% centiles and outliers are plotted as circles outside this range. The numbers of observations in each class are shown.

Measuring the clinical burden posed by *Plasmodium falciparum *infection has been a long-standing challenge for epidemiologists [1-11]. In the absence of complete and accurate health information systems [12], malaria disease burdens have often been modelled from a presumed relationship between infection risk and clinical outcome, extrapolated over mapped proxies of infection risk [4-6,8,9]. These approaches summarize the infection risk and clinical outcome relationship by infection risk classes and thus develop categorical estimates of disease burden. For example, the relationship between *Plasmodium falciparum *malaria prevalence and incidence was used with a categorical global map of malaria endemicity (modified from its historical form [13]) to estimate the clinical burden of malaria globally [10]. The paired prevalence and incidence estimates were grouped for each of the standard endemicity classes [14] (hypoendemic, mesoendemic and hyperendemic and holoendemic combined, see Figure 1) and the median and inter-quartile range used to define the incidence and its error-bounds for each class. When combined with the relevant population surface this procedure provided an estimate of 515 (range 300–660) million clinical episodes of *P. falciparum *malaria in 2002 [10].

A new global map of *P. falciparum *malaria endemicity for 2007 has now been published [15]. This provides a continuous prediction of prevalence (*P. falciparum *parasite rate in the two up to ten year age group, *Pf*PR_2–10_, between 0–100%) for every 5 × 5 km pixel within the stable limits of *P. falciparum *malaria transmission [16]. This map overcomes some of the problems of modified versions of the map created by Lysenko [10]: it is a contemporary measure of global malaria endemicity, based on evidence from a huge repository of parasite rate surveys [17] and implemented in a Bayesian model-based geostatistical framework [18], so that the uncertainty in predictions is represented explicitly [15]. This map therefore, provides a substantially improved spatial platform for future revision of clinical malaria burden estimates.

Since the map now provides estimates of endemicity as a continuous variable [10], in contrast to the historical categorical map [13], it becomes important to investigate the relationship between *P. falciparum *malaria prevalence and incidence. Here, a revised data assembly is presented to better define the continuous relationship between clinical incidence and parasite prevalence. Importantly, the uncertainty in this relationship is modelled and can, therefore, be quantified in future "cartographic" burden estimation initiatives [19,20].

## Methods

### Data assembly

Formal literature searches for data were conducted on PubMed [21] with the search term: "Malaria" [Mesh] AND ("Incidence" [Mesh] OR "Epidemiology" [Mesh] OR "epidemiology" [Subheading]) AND (("1985/01/01" [PDat]:"2008/12/01" [PDat])). These searches were run for the final time on 31 December 2008. These lists were augmented with the results of previous searches [10] and various additional search strategies intended to identify informal literature, including direct approaches to authors. The aim was to identify all active fever surveillance surveys undertaken and reported on since 01 January 1985.

All studies were required to meet the following criteria for inclusion: (i) the study was undertaken since 1985 and thus coincidental with malaria prevalence data used in the global maps of *P. falciparum *endemicity [15]; (ii) surveys were longitudinal and community-based involving prospective active case detection (ACD) of fever cases; (iii) ACD was undertaken at a minimum frequency of fortnightly; (iv) results were presented in a way that allowed the computation of the number of cases and person years of observation; (v) all fever cases were examined for the presence of *P. falciparum *parasites in the peripheral blood; (vi) surveys covered all age groups in the sampled population from birth to adulthood; and (vii) surveys were undertaken over at least one complete 12 month period to account for seasonal variation, or were within a defined (by vector surveillance) period of transmission. A minimum definition of "clinical malaria" was defined as the presence of self-reported or measured fever in a subject who had coincidental *P. falciparum *infection of any parasite density.

Single cross-sectional surveys and surveys where communities were visited monthly and thus likely to miss disease events were not included. Daily surveys were also excluded because these extremely intensive investigations of populations were difficult to separate from the studies' direct influence on local malaria incidence. The frequency of active case detection (ACD) in included studies, therefore, varied between two days and two weeks.

The literature search for data and additional correspondence identified 103 publications and reports with potential data that were reviewed. Data from these studies were extracted and coded according to the method of case ascertainment (ACD, repeat cross-sectional surveys with or without PCD); the frequency of case detection; the case definition used for each study and the number of *P. falciparum *cases identified; the study period and duration and the size of the population studied. From the 103 identified sources, 61 reports fulfilled the combined inclusion criteria (See Additional File 1). Three were excluded because the survey report provided insufficient details on how the survey was conducted, a further nine were excluded because they were not undertaken across a complete calendar year, another sixteen because they were daily, monthly or cross-sectional reports, ten studies because they involved PCD only and four studies for a selection of other reasons. Fourteen surveys were included despite only providing limited details of the surveillance methods, where an informed estimate could be obtained on the frequency of surveillance from parallel literature from the same area or same author (these are indicated in the footnotes in Additional File 1).

Reports capturing more than one village population were disaggregated to represent separate observations where possible. All observations were then geo-positioned using standard techniques outlined elsewhere [17]. In those cases where incidence could be presented for several years, every year of follow up was reported separately. The results of the data abstractions (n = 141 observations) for these epidemiological surveillance studies of *P. falciparum *clinical incidence are shown in Additional File 1. The data are grouped by region, country and location.

### Matching to infection prevalence

A contemporary estimate of *Pf*PR was included with, or obtained through personal communication for, 91 of the 141 clinical incidence observations (Africa+ 16, America 14 and CSE Asia 61). These data are shown in Additional File 1 (column *Pf*PR). Where it was not possible to use the same report to match a congruent estimate of *Pf*PR, the Malaria Atlas Project database [17] was used to search for surveys that were matched as close as possible in time (within five years) and space (within 20 km) to the estimates of clinical incidence. This yielded 23 further matches (Africa+ 4, America 6 and CSE Asia 13). Where this was not possible, the prevalence was predicted using the same database and procedures used to generate a global map of *P. falciparum *endemicity for 2007 [15]. This applied to 27 further points (Africa+ 5, America 0 and CSE Asia 22). These procedures are described in detail elsewhere [15] but, briefly, a Bayesian geostatistical model was constructed that could predict prevalence continuously through space and time by defining a spatio-temporal random field parameterized by a space-time covariance function that included a seasonality component and a mean function that incorporated long-term temporal trends in endemicity and covariates such as the influence of urban and rural areas. The model also incorporated a Bayesian standardization for the age range over which *Pf*PR surveys were conducted allowing all predictions to be made for a common 2–10 year age window. Infection prevalence estimates were therefore provided for this study that matched both the spatial location of each clinical incidence survey and the time period over which it was collected. These data are all shown in Additional File 1 (column *Pf*PR_2–10_). These prevalence predictions were also generated for all incidence studies (Africa+ 25, America 20 and CSE Asia 96) for comparison and are shown in column "*Pf*PR_2–10 _predicted". All subsequent modelling was carried out using three alternative sources of matched infection prevalence values: those from prevalence observations made during, or close in space and time to the incidence surveys only; those resulting from the geostatistical model only; or a combination of the two where the geostatistical predictions were used where suitable observations were not available.

### Experimental approach

The algebraic form of the relationship between average parasite rate and average clinical incidence for *P. falciparum *malaria is not known. However, prior knowledge based on epidemiological evidence does allow certain constraints to be placed on the form of plausible relationships [22]. For example, the relationship must start at the origin (when incidence is zero, prevalence must also be zero), should be relatively smooth (because it is underpinned by a biological mechanism) and its rate of increase should slow and possibly become negative at the highest parasite rates (as the individuals that constitute the population at high endemic levels develop an immunity which enables them to manage the clinical manifestations of infection [22]).

**Figure 2:**
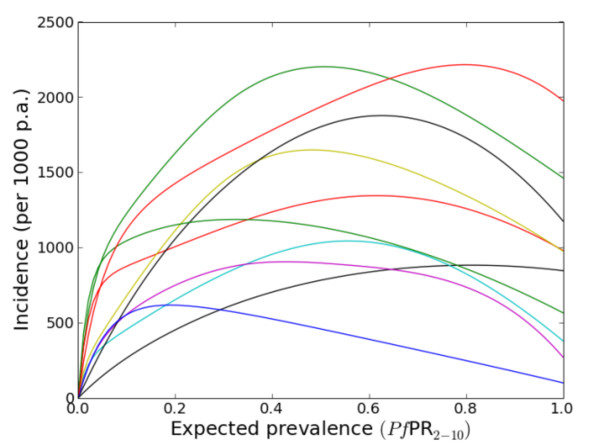
**Draws from the prior of the parasite rate verses clinical incidence relationship**. These curves provide a small yet representative sample of the possible relationships supported by the model. The colours help differentiate the curves.

Faced with this partial knowledge, a typical approach would be to choose between a collection of parametric models, possibly derived using mechanistic considerations (for example [23]), using an information criterion statistic [24]. A key limitation of this approach is that the choice of the model set is *ad-hoc *and can affect substantially the results. In these analyses, an alternative approach was adopted that avoided choosing an arbitrary parametric model. A single, flexible model was defined that could take, or approximate, most reasonable model forms whilst incorporating the basic prior epidemiological constraints. This was achieved with a nonparametric model based on a Gaussian process [25,26]. Standard Bayesian inference was then used to choose between the candidate models when compared to the data. Figure 2 illustrates some examples of possible forms of the parasite rate versus incidence relationship that this model supports. It is important to note that a simple algebraic form cannot be defined for any of them.

**Figure 3:**
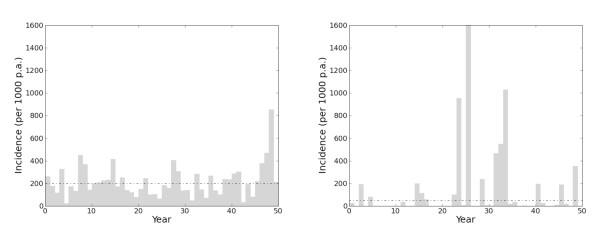
**Typical time-series of annual incidence specified by the temporal volatility model**. The dashed line is the expected incidence (per 1,000 individuals per annum (p.a.)) and the horizontal bars are the observed incidence. The left panel illustrates an "endemic" region of high parasite rate. The incidence in any month is close to the long-term mean, which is relatively high. The right panel shows an "epidemic" region of low parasite rate. The incidence in most months is well below the long-term mean, but occasionally is much greater. The long-term mean in the low parasite rate region is lower than in the high parasite rate region but monthly incidence in the former can occasionally exceed that in the latter.

It is known that opportunistic retrospective assemblies of epidemiological data will display substantial noise, or apparently random variation (See Additional File 1). In the current setting, for a fixed, known value of parasite rate, the noise in the data was attributed to two separate sources: randomness due to the sampling process (clinical incidence estimates recorded over a small survey group will be more uncertain than those from larger surveys) and temporal volatility in incidence (population-wide levels of clinical incidence will naturally fluctuate through time). Temporal volatility is expected to be more severe in areas of low than high endemicity [27]. To incorporate these separate sources of noise and their respective characteristics, a simple sub-model for the unobserved time-series of incidence rates was developed, which was then summed over the surveillance periods to obtain a model for the noise in the observations. This sub-model for the noise can be thought of as an epidemiologically more plausible replacement for the standard negative binomial regression [28]. Examples of the model's behaviour in "endemic" and "epidemic" regions [29] are illustrated schematically in Figure 3.

### Experimental implementation

Expected incidence *f*(*p*) was modelled as a function of age-adjusted parasite rate *p *using a Gaussian process, a very flexible probability distribution for functions. By using a Gaussian process, the overall shape of *f *was constrained to incorporate the prior epidemiological knowledge without imposing any particular parametric model form. A Gaussian process in this context has two components: a mean function (controlling the central tendency of the function at a given value of *p*) and a covariance (controlling the second-order characteristics of the function, such as its smoothness).

The Gaussian covariance model [26] was used to ensure that *f *was infinitely differentiable, meaning not rough. This smoothness was considered desirable because the biological mechanisms causing these relationships and those forms chosen by most parametric models would also be smooth. The mean function of *f *was modelled using the simple parametric model *E*[*f*(*p*)] = *a*(1 - exp(-*bp*)) where *a *and *b *are unknown and assigned uniform priors between 0 and 100, and 0.1 and 100, respectively. The Gaussian process distribution was conditioned so that *f*(0) = 0, in other words incidence was constrained to be zero when parasite rate was zero. Values of 0.6 and 1 were chosen *a priori *for the range and sill parameters of *f*. It would have been preferable to infer them with the other parameters, but this more flexible model was prohibitively difficult to fit. These values were chosen because they produced a range of curve shapes for *f *that were judged to be reasonable.

**Figure 4:**
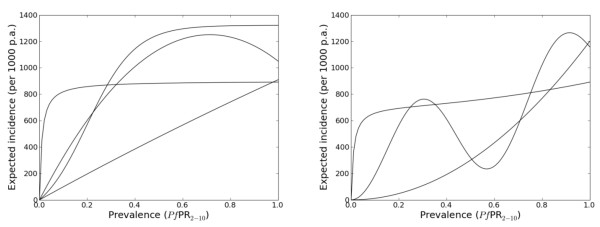
**Possible shapes for the relationship between parasite rate and expected incidence**. The relationship between expected incidence per 1000 individuals per annum (p.a.) and prevalence was constrained to be concave down at a parasite rate of 1 and to have at most one inflection point. The curves in the left-hand panel are examples of allowable relationships by these criteria, and the curves in the right-hand panel are examples of relationships that are not allowed.

Three prior constraints were imposed on *f*. Firstly, *f *was constrained to be concave down at *x *= 1, *i.e*. *f" *(1) < 0. This ensured the rate of increase in incidence would eventually slow or reverse with increasing prevalence. Secondly, *f *was constrained to have at most one inflection point between 0 and 1, that is, *f" *could change sign only once. This ensured a relatively simple model form without multiple peaks and troughs. Thirdly, *f *was constrained to be positive on the interval (0, 1]. The effects of these series of constraints are illustrated in Figure 4.

Temporal volatility in incidence was modelled using a gamma process [18]. Heuristically, this is a stochastic process that produces intermittent "spikes" punctuating periods of quiescence. In high parasite rate (PR) areas incidence fluctuates little between years, whilst in low-PR areas incidence is highly volatile, with a background of low incidence punctuated by sporadic high incidence events (occasional outbreaks/epidemics) [30,31]. These characteristics of temporal volatility were modelled as follows. Firstly, the amount of volatility *r *was modelled as a quadratic function of prevalence:

(1)

When *r *is low, volatility will be high and *vice versa*. Uninformative priors were used for the parameters of *r *subject to the constraint that *r' *≥ 0 on (0, 1]. Secondly, a theoretical time series of incidence *a *was modeled using a gamma process [32] parameterized by the volatility *r *and the mean incidence *f*(*p*_*j*_):

(2)

Given the time series of incidence *a*_*j*_, observed cases were modelled as outcomes of a Poisson process [32]. Formally, in location *j*, the times of infections *τ *for each individual *i *were modelled as:

(3)

These three statements (equations 1 to 3) together formed an explicit model for the timings of individual clinical events. However, this model can easily be aggregated to obtain a model for the total number of cases observed by each study.

The influence of observation frequency on fever detection has been described elsewhere [33], so the time intervals of surveys were standardized to approximate weekly surveillance where required. When ACD incidence was described without accompanying passive case detection (PCD), frequency was scaled to every seven days by multiplying *t*_*j *_by the appropriate factor: fortnightly surveillance by 2.000 and surveillance conducted every ten, three and two days by 1.4002, 0.429, 0.286 respectively. For studies that were conducted alongside PCD, *t*_*j *_was unadjusted assuming missed cases would be detected passively.

It was assumed that *P. falciparum *infections will tend to clear within a week, so that weekly active surveillance will catch essentially all cases whereas fortnightly active surveillance will catch cases with probability 0.5. Passive surveillance, however, was assumed to catch all cases even if the interval of surveillance is greater than fortnightly. It was also assumed that surveillance that is more frequent than weekly will double-count some cases.

For surveillance program *j*, which involves *n*_*j *_individuals, lasts *t*_*j *_years and surveys at intervals of *d*_*j *_days, the model for total cases *y*_*j *_obtained by aggregating models (1–3) was:

(4)

This likelihood model and the Gaussian process model for *f*, together with uniform priors for the scalar parameters with appropriate constraints (such as positivity), formed a Bayesian probability model for the data. The model was fitted using Markov chain Monte Carlo [34] using the open-source Bayesian analysis package PyMC [35]. The code is freely available upon request.

### Spatial dependence

If the prevalence and incidence relationship varied spatially within a given region, then the use of a single model for all areas in that region would lead to worse model fit in some areas than others. This, in turn, would be manifested as spatial dependence in the model residuals [18,36,37]. To investigate this possibility, sample semivariograms of the mean deviance residuals in each were examined.

## Results

Comparison of results of the predicted prevalence and incidence relationship obtained using the three alternative sources of matched *Pf*PR prevalence values (observed contemporarily to the incidence surveys, predicted with the geostatistical model, or a combination of the two) revealed very little difference. The decision was made, therefore, to use prevalence values predicted by the geostatistical model as the matched value for all incidence surveys, regardless of whether they had an observed prevalence value available, and these are the results presented here. The geostatistical model was preferred because prevalence values were matched to incidence surveys exactly in space and time and were age-standardized. An additional advantage was that these modelled prevalences will form the basis for future burden estimates so their use in this study provides a consistency with future work. The results obtained using the alternative matched prevalence values are available on request.

**Figure 5:**
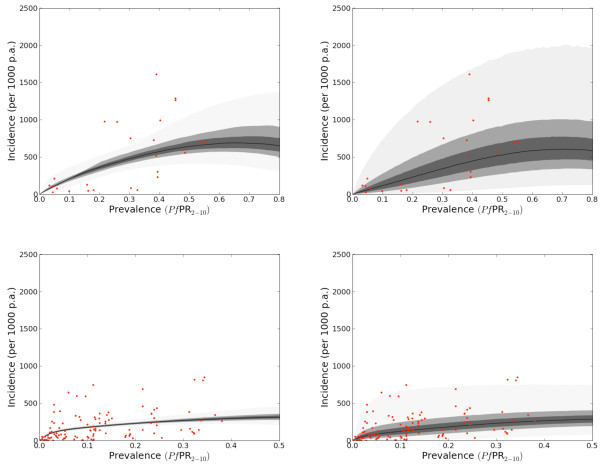
**Relationship between parasite rate and incidence**. Left: the posterior distribution of expected population-wide incidence (per 1000 individuals per annum (p.a.)) against prevalence. This relationship would describe the observed incidence if surveillance were conducted for a very long time. Right: the predictive distribution of actual population-wide incidence per 1000 individuals in any given year. The right-hand panels take temporal volatility into account, therefore, whereas the left-hand panels average it out. The top row shows the Africa+ region (n = 25) and the bottom the CSE Asia and the Americas regions combined (n = 116). The data points are the red dots, the black line is the median and the 0.25, 0.5 and 0.95 credible intervals centred on the median are shown in shades of progressively lighter grey.

The initial intention was to model each biogeographically, entomologically and epidemiologically distinct [38-40] global region (the Americas, Africa+ (Africa, Yemen and Saudi Arabia) and CSE Asia (Central and South East Asia) independently. There were so few data for the Americas, however, that they were merged with those from CSE Asia, assuming that the epidemiology of *P. falciparum *was relatively more similar between these regions than between the Americas and the Africa+ region. The results are shown in Figure 5.

The left-hand panels show the posterior distribution of *f*, the expected incidence. The right-hand panels show the predictive distribution of observed incidence *y *for a hypothetical future survey that would sample 1000 individuals weekly for two years. These "predictive panels" are a better indication of the credible intervals that would be used in burden estimation in a small area because the "posterior panels" do not incorporate temporal volatility. The "posterior panels" predict the average incidence in a region over a period of many years, rather than in any particular year. In other words, they average out the natural temporal variation that is regularly observed from year to year in malaria incidence, whereas the "predictive panels" incorporate this variation. These considerations are explored further in the discussion.

In each region, incidence increased slowly and smoothly as a function of prevalence; reaching about 250 and 500 cases per thousand of the population *per annum *at parasite prevalences above 40% in the Americas and CSE Asia combined region and the Africa+ region respectively. As expected, incidence is highly variable for any given prevalence. In the Africa+ region, for example, the predicted 95% credible interval for the number of observed clinical attacks, given that 1000 individuals from a population with a parasite rate of 30% will be sampled weekly for two years, extends from less than 20 to about 1500. This range may seem huge, but it is not unrepresentative of the data: the four observations for which PR is between 10% and 25% had scaled incidences of 127.7, 53.8, 43.1 and 975.6. In addition, the scarcity of data in the Africa+ region causes relatively large parameter uncertainty. In CSE Asia and the Americas, the analogous interval extends from about 50 to more than 700. While many more data points are available in this region, they are clearly very noisy. Scaled incidence for points with parasite rates between 10% and 25% ranged from about 3 to 750.

**Figure 6:**
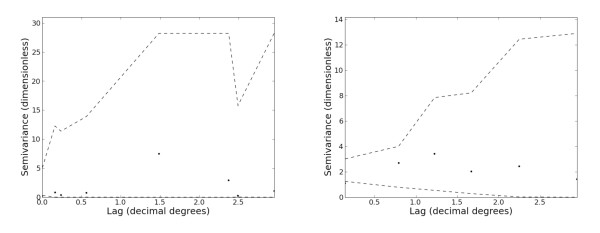
**Spatial semivariograms of model residuals**. Sample semivariograms of deviance residuals estimated at discrete lags (circles) and compared to a Monte Carlo null envelope (dashed lines) representing the range of values expected by chance in the absence of spatial autocorrelation. An estimated value falling outside the null envelope is indicative of significant spatial structure in the residuals at that lag. Shown are plots for the Africa+ region (left) and the combined Central and South East Asia and Americas regions (right).

There was some evidence of weak structure in the combined CSE Asia and Americas semivariograms plots (Figure 6) but this was not statistically significant and hence not of a level that would necessitate correcting the credible interval defined here. No spatial dependence could be determined in the noisy variograms from the Africa+ region.

## Discussion

This study provides the first example of a nonparametric Bayesian inference of a functional relationship in epidemiology, although Munch *et al *[41] used a similar approach in a comparable model selection problem in fishery science. Moreover, this work also models for the first time the continuous relationship between paired prevalence and incidence in communities.

### Regional variation

In each region, incidence increased gradually and smoothly (as specified by the model) as a function of prevalence, but was highly variable for any given prevalence. These relationships had similar forms in the Africa+ and the combined America and CSE Asia regions. The rate of increase in prevalence in the Africa+ region was higher however, reaching levels of around 500 cases per thousand of the population *per annum *at prevalences above 40% compared to approximately 250 cases per thousand of the population *per annum *in the combined America and CSE Asia data.

There are many potential reasons as to why these relationships may show regional variation, including: differences in the dominant vector species [39,42], the impact of drug resistance on recrudescent clinical attacks [43], the possible modification of *P. falciparum *clinical outcomes in areas of *Plasmodium vivax *co-infection [44,45] and the genetic contribution to disease risks as part of regionally defined inherited blood disorders [46].

### Effect of the time period modelled on credible intervals

A critical component of the modelling approach outlined is the incorporation of temporal volatility in incidence rate: its natural tendency to fluctuate independently of changes in prevalence. An important implication of this mechanism is illustrated in Figure 5 in which the left panels show the average incidence if a population were held at a constant parasite rate for a very long time, and the right panels show the average incidence over a year. Although it is tempting to consider the left-hand panels as illustrating the "underlying" relationship that remains after the noise in the data has been filtered out, the right-hand panels are the correct ones to associate with predictions for a given year in a single population. Intuitively, this can also be understood because the credible intervals of the right-hand panels (Figure 5) match more closely the range of the observed data. The noisy predictions reflect the empirical fact that the situation at any given time in a region may deviate significantly from any "typical" transmission pattern that conventional wisdom dictates there.

### Caveats

In a departure from standard epidemiological practice, the credible intervals derived capture uncertainty in the long-term relationship, as well as uncertainty expected in future observations due to temporal volatility. Rather than considering the randomness in the data to be a nuisance that obscures the true malaria dynamics, it has been modelled as an essential component of the predictive statistical method.

In addition to assumptions about the relationship between parasite rate and expected incidence, these results depend on assumptions about the temporal volatility. Firstly, it was assumed that volatility decreased with increasing average parasite rate. Examination of the posterior distribution of the volatility function *r *suggested that this constraint did not restrict artificially the values fitted by the model (this was not shown). Secondly, it was assumed that factors such as age and immunity that affect individuals' probability of a clinical episode independently from prevalence did not differ systematically between communities.

The model also assumed that, given average parasite rate, incidence at different times and places was independent. In other words, all spatiotemporal correlation in incidence was driven by that in the underlying parasite rate. This assumption is likely to have implications for predictions of burden over extended spatiotemporal regions and should be carefully examined when more relevant data are available. Furthermore, we have not considered the possible influence on the prevalence-incidence relationship of morbidity from other sources such as co-infection [47]. The absence of clear spatial autocorrelation in the model residuals suggests that, if any such effects were induced by a secondary spatially varying condition, these effects were weak.

### Future work

This study has focussed on describing an empirical relationship to support burden estimation initiatives but there is clearly a significant opportunity for extending mathematical modelling work [23,48,49] in this area to help guide thinking about plausible biological mechanisms. This study has been reliant on a series of surveys conducted by a range of authors, in a range of countries with very different objectives. It is not inconceivable that a series of planned systematic prospective studies, for example a stratified sample of new paired incidence and prevalence measures along a gradient of transmission in each continent, would reduce substantially the uncertainty associated with this relationship.

This work is informative about the nature of the relationship between incidence and prevalence but also provides an important step in enabling revisions of the global clinical burden of *P. falciparum *malaria. The Malaria Atlas Project [19,20] is currently developing the computational tools needed to draw samples from the joint predictive distribution of the global endemicity surface (also known as "conditional simulations") [15]. The statistical model presented here was developed specifically for the purpose of converting these samples to corresponding samples of the global incidence surface, and thence to samples from the predictive distribution of burden in any geographical area.

**Figure 7:**
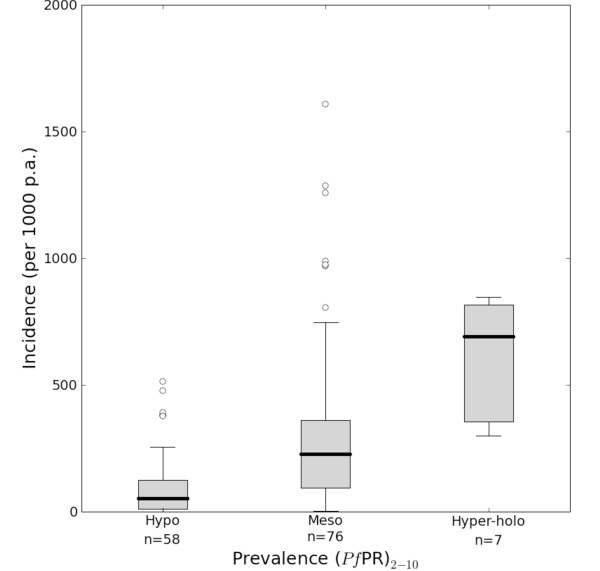
**Annual clinical incidence of *P. falciparum *per 1,000 population in hypoendemic, mesoendemic and combined hyperendemic and holoendemic prevalence conditions**. The box indicates the inter-quartile range (25% and 75%) and the thick line within the box represents the median. The whiskers represent the 2.5% and 97.5% centiles and outliers are plotted as circles outside this range. The number of observations in each class is shown.

It is hard to anticipate how these results might affect estimates of the global burden of malaria and so we conclude with three observations that might be illustrative of the direction and influences. First, if the summaries of these incidence data are conducted in the same way as previous burden estimates [10], the median incidences described for each endemicity class are very similar (compare Figure 1 and Figure 7), but the variance around these estimates, described by the inter-quartile ranges, much less. Second, much of the contemporary endemicity in the world [15] is described by much lower prevalence than would be suggested by inspecting historical maps [13], with median prevalence values for the America, Africa and CSE Asia regions of approximately 2, 33 and 10% respectively. This lower contemporary description of endemicity would suggest a concurrent lowering in the morbidity estimate compared to earlier attempts. Moreover, large areas of each region are at predicted prevalence levels where the credible intervals for clinical incidence are relatively small, which augurs well for improving the uncertainty in future burden estimates. Third, independent of these changes, population growth [50,51] will have continued apace since the last estimates in 2002 and therefore increase population denominators included in the burden calculations. Ongoing efforts are focussed on resolving these interacting influences and providing revised "cartographic" clinical burden estimates for 2007.

## Competing interests

The authors declare that they have no competing interests.

## Authors' contributions

SIH conceived the study and participated in its design and coordination. EAO assembled the bulk of the incidence data with assistance from CAG and SKS. APP and PWG devised the methods. APP implemented the analyses. SIH wrote the first draft of the manuscript. All authors participated in the interpretation of results and in the writing and editing of the manuscript.

## Supplementary Material

Additional File 1 **Matching *Plasmodium falciparum *clinical incidence and prevalence data**. A fully referenced table of the *Plasmodium falciparum *clinical incidence and matched age-stratified *Plasmodium falciparum *parasite data. Click here for file

## References

[B1] Bruce-Chwatt LJ (1952). Malaria in African infants and children in Southern Nigeria. Ann Trop Med Parasitol.

[B2] Carter R, Mendis KN (2002). Evolutionary and historical aspects of the burden of malaria. Clin Microbiol Rev.

[B3] Greenwood BM (1990). Population at risk. Parasitol Today.

[B4] Hay SI, Guerra CA, Tatem AJ, Atkinson PM, Snow RW (2005). Urbanization, malaria transmission and disease burden in Africa. Nat Rev Microbiol.

[B5] Roca-Feltrer A, Carneiro I, Armstrong Schellenberg JR (2008). Estimates of the burden of malaria morbidity in Africa in children under the age of 5 years. Trop Med Int Health.

[B6] Rowe AK, Rowe SY, Snow RW, Korenromp EL, Schellenberg JRA, Stein C, Nahlen BL, Bryce J, Black RE, Steketee RW (2006). The burden of malaria mortality among African children in the year 2000. Int J Epidemiol.

[B7] Schwartlander B (1997). Global burden of disease. Lancet.

[B8] Snow RW, Craig M, Deichmann U, Marsh K (1999). Estimating mortality, morbidity and disability due to malaria among Africa's non-pregnant population. Bull World Health Organ.

[B9] Snow RW, Craig MH, Newton CRJC, Steketee RW (2003). The public health burden of *Plasmodium falciparum *malaria in Africa: deriving the numbers. Working Paper No11, Disease Control Priorities Project.

[B10] Snow RW, Guerra CA, Noor AM, Myint HY, Hay SI (2005). The global distribution of clinical episodes of *Plasmodium falciparum *malaria. Nature.

[B11] Sturchler D (1989). How much malaria is there worldwide?. Parasitol Today.

[B12] Gething PW, Noor AM, Gikandi PW, Ogara EAA, Hay SI, Nixon MS, Snow RW, Atkinson PM (2006). Improving imperfect data from health management information systems in Africa using space-time geostatistics. PLoS Med.

[B13] Lysenko AJ, Semashko IN, Lebedew AW (1968). Geography of malaria. A medico-geographic profile of an ancient disease [in Russian]. Itogi Nauki: Medicinskaja Geografija.

[B14] Metselaar D, Van Thiel PH (1959). Classification of malaria. Trop Geogr Med.

[B15] Hay SI, Guerra CA, Gething PW, Patil AP, Tatem AJ, Noor AM, Kabaria CW, Manh BH, Elyazar IRF, Brooker SJ, Smith DL, Moyeed RA, Snow RW (2009). A world malaria map: *Plasmodium falciparum *endemicity in 2007. PLoS Med.

[B16] Guerra CA, Gikandi PW, Tatem AJ, Noor AM, Smith DL, Hay SI, Snow RW (2008). The limits and intensity of *Plasmodium falciparum *transmission: implications for malaria control and elimination worldwide. PLoS Med.

[B17] Guerra CA, Hay SI, Lucioparedes LS, Gikandi PW, Tatem AJ, Noor AM, Snow RW (2007). Assembling a global database of malaria parasite prevalence for the Malaria Atlas Project. Malar J.

[B18] Diggle PJ, Ribeiro PJ (2007). Model-based geostatistics.

[B19] Hay SI (2009). The Malaria Atlas Project (MAP). http://www.map.ox.ac.uk.

[B20] Hay SI, Snow RW (2006). The Malaria Atlas Project: developing global maps of malaria risk. PLoS Med.

[B21] Anonymous PubMed: A service of the U.S. National Library of Medicine and the National Institutes of Health. http://www.ncbi.nlm.nih.gov/sites/entrez.

[B22] Snow RW, Marsh K (2002). The consequences of reducing transmission of *Plasmodium falciparum *in Africa. Adv Parasitol.

[B23] Smith DL, Dushoff J, Snow RW, Hay SI (2005). The entomological inoculation rate and *Plasmodium falciparum *infection in African children. Nature.

[B24] Gelman A, Carlin JB, Stern HS (2003). Bayesian data analysis. Texts in Statistical Science.

[B25] MacKay DJC (1998). Introduction to Gaussian Processes. Neural networks and machine learning. NATO advanced study institute on generalization in neural networks and machine learning, Cambridge, 4–15 August 1997. NATO.

[B26] Banerjee S, Carlin BP, Gelfand AE (2004). Hierarchical modeling and analysis for spatial data. Monographs on Statistics and Applied Probability 101.

[B27] Hay SI, Myers MF, Burke DS, Vaughn DW, Endy T, Ananda N, Shanks GD, Snow RW, Rogers DJ (2000). Etiology of interepidemic periods of mosquito-borne disease. Proc Natl Acad Sci USA.

[B28] Hilbe JM (2007). Negative binomial regression.

[B29] Nájera JA, Kouznetsov RL, Delacollete C (1998). Malaria epidemics. Detection and control, forecasting and prevention. WHO/MAL/98.1084.

[B30] Hay SI, Simba M, Busolo M, Noor AM, Guyatt HL, Ochola SA, Snow RW (2002). Defining and detecting malaria epidemics in the highlands of western Kenya. Emerg Infect Dis.

[B31] Hay SI, Were EC, Renshaw M, Noor AM, Ochola SA, Olusanmi L, Alipui N, Snow RW (2003). Forecasting, warning, and detection of malaria epidemics: a case study. Lancet.

[B32] Møller J, Waagepetersen RP (2004). Statistical inference and simulation for spatial point processes. Monographs on Statistics and Applied Probability 100.

[B33] Snow RW, Menon A, Greenwood BM (1989). Measuring morbidity from malaria. Ann Trop Med Parasitol.

[B34] Gilks WR, Spiegelhalter DJ (1999). Markov Chain Monte Carlo in practice. Interdisciplinary Statistics.

[B35] Patil A, Huard D, Fonnesbeck C (2008). PyMC: Markov Chain Monte Carlo for Python, version 2.0. Journal of Statistical Software.

[B36] Diggle PJ, Tawn JA, Moyeed RA (1998). Model-based geostatistics. J Roy Stat Soc C-App.

[B37] Goovaerts P (1997). Geostatistics for natural resource evaluation.

[B38] Hay SI, Sinka ME, Tatem AJ, Patil AP, Guerra CA, Okara RM, Howes RE, Kabaria CK, Bangs M, Chareonviriyaphap T, Godfray HCJ, Harbach R, Hemingway J, Mbogo CM, Rogers DJ, Rubio-Palis Y (2009). Developing global maps of the dominant *Anopheles *vectors of human malaria. PLoS Med.

[B39] Macdonald G (1957). Local features of malaria. The epidemiology and control of malaria.

[B40] Mouchet J, Carnevale P, Coosemans M, Julvez J, Manguin S, Richard-Lenoble D, Sircoulon J (2004). Paludisme et grandes régions biogéographiques. Biodiversité du paludisme dans le monde.

[B41] Munch SB, Kottas A, Mangel M (2005). Bayesian nonparametric analysis of stock-recruitment relationships. Can J Fish Aquat Sci.

[B42] Mouchet J, Carnevale P, Coosemans M, Julvez J, Manguin S, Richard-Lenoble D, Sircoulon J (2004). Biodiversité du paludisme dans le monde.

[B43] Talisuna AO, Bloland P, D'Alessandro U (2004). History, dynamics, and public health importance of malaria parasite resistance. Clin Microbiol Rev.

[B44] Gunewardena DM, Carter R, Mendis KN (1994). Patterns of acquired anti-malarial immunity in Sri Lanka. Mem Inst Oswaldo Cruz.

[B45] Maitland K, Williams TN, Newbold CI (1997). *Plasmodium vivax *and *P. falciparum *: biological interactions and the possibility of cross-species immunity. Parasitol Today.

[B46] Weatherall D, Akinyanju O, Fucharoen S, Olivieri N, Musgrove P, Jamison DT, Breman JG, Measham AR, Alleyne G, Claeson M, Evans DB, Jha P, Mills A, Musgrove P (2006). Inherited disorders of hemoglobin. Disease control priorities in developing countries.

[B47] Brooker S, Clements ACA, Hotez PJ, Hay SI, Tatem AJ, Bundy DAP, Snow RW (2006). The co-distribution of *Plasmodium falciparum *and hookworm among African schoolchildren. Malar J.

[B48] Smith DL, McKenzie FE (2004). Statics and dynamics of malaria infection in *Anopheles *mosquitoes. Malar J.

[B49] Smith DL, McKenzie FE, Snow RW, Hay SI (2007). Revisiting the basic reproductive number for malaria and its implications for malaria control. PLoS Biol.

[B50] Balk DL, Deichmann U, Yetman G, Pozzi F, Hay SI, Nelson A (2006). Determining global population distribution: methods, applications and data. Adv Parasitol.

[B51] Hay SI, Noor AM, Nelson A, Tatem AJ (2005). The accuracy of human population maps for public health application. Trop Med Int Health.

