# Supplementary material for: Defining the relationship between Plasmodium falciparum parasite rate and clinical disease: statistical models for disease burden estimation
Source: Malar J. 2009 Aug 5;8:186. doi: 10.1186/1475-2875-8-186 (PMC2746234; doi:10.1186/1475-2875-8-186)
Supplement: Additional File 1 — Matching Plasmodium falciparum clinical incidence and prevalence data. A fully referenced table of the Plasmodium falciparum clinical incidence and matched age-stratified Plasmodium falciparum parasite data. [file 1475-2875-8-186-S1.doc]

**Matching *Plasmodium falciparum* clinical incidence and prevalence data**

| **Region** | **Country** | **ACD location** | **Latitude** | **Longitude** | **Start year** | **End year** | **Freq of ACD** | **PCD** | ***d*** | **PYO** | ***γ*** | ***Pf*PR** | ***Pf*PR_2-10_** | ***Pf*PR_2-10_ predicted** | **Ref ACD** | **Ref *Pf*PR** |
| --- | --- | --- | --- | --- | --- | --- | --- | --- | --- | --- | --- | --- | --- | --- | --- | --- |
| Africa+ | Kenya | Kipsamoite | 0.3229 | 34.9991 | 2001 | 2001 | Weekly | Y | 116 | 2913 | 39.821 | 1.316 | 1.725 | 9.897 | [1] | [2] |
| Africa+ | Kenya | Kipsamoite | 0.3229 | 34.9991 | 2002 | 2002 | Weekly | Y | 388 | 3038 | 127.716 | 7.895 | 10.348 | 15.851 | [1] | [2] |
| Africa+ | Kenya | Kipsamoite | 0.3229 | 34.9991 | 2003 | 2003 | Weekly | Y | 167 | 3094 | 53.975 |  | 17.964 | 17.964 | [1] | [3] |
| Africa+ | Kenya | Kipsamoite | 0.3229 | 34.9991 | 2004 | 2004 | Weekly | Y | 124 | 2877 | 43.100 |  | 16.271 | 16.271 | [1] | [3] |
| Africa+ | Kenya | Ngilai | 1.2206 | 37.3264 | 2001 | 2004 | Weekly | Y | 13 | 238 | 54.622 |  | 32.662 | 32.662 | [4] | [3] |
| Africa+ | Kenya | Ngerenya | -3.5153 | 39.8376 | 1999 | 2000 | Weekly | Y | 583 | 779 | 748.290 | 21.844 | 22.027 | 30.289 | [5-7] | [5] |
| Africa+ | Kenya | Ngerenya | -3.5153 | 39.8376 | 2000 | 2001 | Weekly | Y | 882 | 910 | 968.794 | 28.399 | 28.636 | 25.909 | [5-7] | [5] |
| Africa+ | Kenya | Chonyi | -3.7231 | 39.7249 | 2000 | 2001 | Weekly | Y | 609 | 843 | 722.180 | 38.348 | 38.669 | 38.124 | [5-7] | [5] |
| Africa+ | Kenya | Chonyi | -3.7231 | 39.7249 | 1999 | 2000 | Weekly | Y | 415 | 746 | 556.420 | 42.805 | 43.163 | 48.585 | [5-7] | [5] |
| Africa+ | Madagascar | Ankazobe | -18.3160 | 47.1180 | 1993 | 1996 | Every 2nd day | Y | 949 | 3316 | 81.768 | 14.321 | 18.771 | 30.412 | [8] | [8] |
| Africa+ | Mali | Donéguébougou | 12.8010 | -7.9840 | 1999 | 1999 | Weekly | Y | 262 | 379 | 691.293 | 54.086 | 59.206 | 55.307 | [9] | [9] |
| Africa+ | Mali | Donéguébougou | 12.8010 | -7.9840 | 2000 | 2000 | Weekly | Y | 271 | 389 | 697.375 | 48.314 | 52.887 | 53.921 | [9] | [9] |
| Africa+ | Mali | Sotuba | 12.6490 | -7.9340 | 1999 | 1999 | Weekly | Y | 379 | 383 | 989.040 | 15.061 | 16.487 | 40.318 | [9] | [9] |
| Africa+ | Mali | Sotuba | 12.6490 | -7.9340 | 2000 | 2000 | Weekly | Y | 197 | 383 | 514.092 | 6.074 | 6.649 | 38.910 | [9] | [9] |
| Africa+ | Mali | Koro (Dogon) | 14.5111 | -3.0705 | 1999 | 2000 | Every 3rd day | Y | 1390 | 1970 | 302.393 | 24.030 | 31.497 | 39.318 | [10] | [10] |
| Africa+ | Mali | Koro (Fulani) | 14.5111 | -3.0705 | 1999 | 2000 | Every 3rd day | Y | 292 | 552 | 226.708 | 14.241 | 18.667 | 39.319 | [10] | [10] |
| Africa+ | Mali | Bandiagara (control) | 14.3440 | -3.6060 | 1999 | 1999 | Weekly | Y | 199 | 158 | 1261.090 | 40.909 | 42.811 | 45.333 | [11] | [12] |
| Africa+ | Mali | Bandiagara (SP Rx) | 14.3440 | -3.6060 | 1999 | 1999 | Weekly | Y | 202 | 157 | 1284.170 | 40.909 | 42.811 | 45.378 | [11] | [12] |
| Africa+ | Senegal | Dakar | 14.7170 | -17.4500 | 1996 | 1997 | Weekly | N | 26 | 1067 | 24.367 | 1.398 | 1.398 | 4.421 | [13] | [13] |
| Africa+ | Senegal | Ndiop | 13.6830 | -16.3830 | 1993 | 1994 | Weekly | Y | 565 | 351 | 1609.687 | 29.912 | 30.930 | 38.956 | [14] | [14] |
| Africa+ | Senegal | Barkedji | 15.1700 | -14.5300 | 1994 | 1995 | Every 10 days | N | 84 | 123 | 975.610 |  | 21.743 | 21.743 | [15] | [3] |
| Africa+ | South Africa | Ndumu and Makanis area | -27.1000 | 32.2110 | 1996 | 1996 | Weekly* | Y | 2725 | 13099 | 208.031 |  | 4.959 | 4.959 | [16] | [3] |
| Africa+ | Sudan | Daraweesh | 13.9330 | 35.4980 | 1993 | 1993 | Every 2nd day | Y | 158 | 415 | 108.778 | 8.854 | 11.605 | 4.207 | [17] | [17] |
| Africa+ | Sudan | Daraweesh | 13.9330 | 35.4980 | 1994 | 1994 | Every 2nd day | Y | 176 | 432 | 116.402 | 8.854 | 11.605 | 3.266 | [17] | [17] |
| Africa+ | Sudan | Daraweesh | 13.9330 | 35.4980 | 1995 | 1995 | Every 2nd day | Y | 108 | 421 | 73.295 | 1.751 | 2.295 | 5.775 | [17] | [17] |
| America | Brazil | Urupa | -9.0860 | -63.6680 | 1996 | 1996 | Weekly* | Y | 4 | 145 | 27.586 | 1.379 | 1.808 | 5.166 | [18] | [19] |
| America | Brazil | Ramal do Granada | -10.2416 | -67.2083 | 2004 | 2005 | Every 2nd day | Y | 108 | 636 | 48.498 | 0.720 | 0.989 | 0.481 | [20] | [21] |
| America | Brazil | Urupa | -9.0860 | -63.6680 | 1991 | 1992 | Weekly | Y | 64 | 168 | 380.952 | 0.792 | 1.038 | 2.967 | [19] | [19] |
| America | Brazil | Urupa | -9.0860 | -63.6680 | 1991 | 1992 | Weekly | Y | 69 | 144 | 478.890 | 0.792 | 1.038 | 2.969 | [22] | [19] |
| America | Brazil | Portucheulo | -8.6080 | -63.6100 | 1998 | 1999 | Every 3rd day | Y | 28 | 175 | 68.571 | 5.480 | 7.183 | 4.013 | [23] | [23] |
| America | Brazil | Leonislandia | -10.2770 | -54.9840 | 1996 | 1997 | Weekly* | Y | 22 | 521 | 42.226 | 2.400 | 3.146 | 7.871 | [24] | [24] |
| America | Brazil | Vila Candelaria | -8.7855 | -63.9177 | 2001 | 2001 | Weekly | Y | 22 | 270 | 81.481 | 6.129 | 8.034 | 4.499 | [25] | [25] |
| America | Brazil | Vila Candelaria | -8.7855 | -63.9177 | 2002 | 2002 | Weekly | Y | 22 | 270 | 81.481 | 9.322 | 12.219 | 5.300 | [25] | [25] |
| America | Guatemala | Los Amates | 15.2570 | -89.1010 | 1990 | 1991 | Fortnightly | Y | 11 | 350 | 31.429 | 0.391 | 0.512 | 2.114 | [26] | [26] |
| America | Peru | Tambogrande/Bellavista (Dry) | -4.9500 | -80.5340 | 1996 | 1997 | Weekly | N | 6 | 4510 | 1.330 | 0.210 | 0.275 | 1.047 | [27] | [27] |
| America | Peru | Tambogrande/Bellavista II (Int) | -4.9170 | -80.5170 | 1996 | 1997 | Weekly | N | 42 | 4598 | 9.134 | 0.405 | 0.531 | 1.077 | [27] | [27] |
| America | Peru | Tambogrande/Bellavista III (Green) | -4.8700 | -80.5500 | 1996 | 1997 | Weekly | N | 175 | 3324 | 52.647 | 1.280 | 1.678 | 1.140 | [27] | [27] |
| America | Peru | Padre Cocha | -3.6986 | -73.2775 | 1997 | 1998 | Weekly* | Y | 232 | 1400 | 165.714 | 1.300 | 1.704 | 4.120 | [28] | [28] |
| America | Venezuela | Las Majadas (non-vaccine) | 7.6330 | -64.8340 | 1988 | 1989 | Fortnightly | Y | 49 | 941 | 52.084 | 0.441 | 0.743 | 3.531 | [29] | [30] |
| America | Venezuela | Las Majadas (non-vaccine) | 7.6330 | -64.8340 | 1989 | 1990 | Fortnightly | Y | 56 | 825 | 67.852 | 0.441 | 0.743 | 3.378 | [29] | [30] |
| America | Venezuela | Las Majadas | 7.6330 | -64.8340 | 1988 | 1989 | Fortnightly | Y | 89 | 941 | 94.601 | 0.441 | 0.743 | 3.523 | [29] | [30] |
| America | Venezuela | Ocamo & Mavaca (Int) | 2.5088 | -65.1461 | 1999 | 2000 | Fortnightly | Y | 73 | 835 | 87.415 | 1.024 | 1.331 | 3.082 | [31] | [31] |
| America | Venezuela | Ocamo & Mavaca | 2.7690 | -65.2031 | 1999 | 2000 | Fortnightly | Y | 108 | 917 | 117.814 | 4.217 | 5.480 | 2.495 | [31] | [31] |
| America | Venezuela | Coyoweteri & Coshiloweteri (main) | 2.5470 | -64.7680 | 1993 | 1994 | Weekly* | Y | 32 | 625 | 51.173 | 6.600 | 8.651 | 5.503 | [32] | [32] |
| America | Venezuela | Coyoweteri & Coshiloweteri (remote) | 3.8550 | -65.6950 | 1993 | 1994 | Weekly* | Y | 32 | 1123 | 28.504 | 6.600 | 8.651 | 5.289 | [32] | [32] |
| CSE Asia | China | Bainan (C) | 23.0650 | 105.8310 | 1990 | 1992 | Weekly* | N | 30 | 12308 | 2.437 |  | 0.920 | 0.920 | [33] | [3] |
| CSE Asia | China | Baisen | 23.1576 | 105.6666 | 1990 | 1991 | Weekly* | N | 11 | 3565 | 3.086 | 4.600 | 6.029 | 0.881 | [33] | [33] |
| CSE Asia | China | Dayouza (b4 intervention) | 25.4140 | 107.8800 | 1990 | 1990 | Weekly* | N | 101 | 2889 | 34.960 | 5.300 | 6.947 | 3.815 | [33] | [33] |
| CSE Asia | China | Genban (C) | 25.3710 | 107.8560 | 1990 | 1991 | Weekly* | N | 188 | 12423 | 15.133 | 9.000 | 11.797 | 4.854 | [33] | [33] |
| CSE Asia | China | Nalong & Pingmon (b4 int) | 23.3234 | 105.7117 | 1990 | 1990 | Weekly* | N | 29 | 18920 | 1.533 | 4.700 | 6.160 | 1.055 | [33] | [33] |
| CSE Asia | India | Anandpur | 28.5060 | 77.6170 | 2004 | 2006 | Fortnightly | N | 67 | 2000 | 67.000 | 5.636 | 7.387 | 1.707 | [34] | [34] |
| CSE Asia | India | Beel Akbarpur | 28.4880 | 77.6090 | 2004 | 2006 | Fortnightly | N | 18 | 3600 | 10.000 | 1.707 | 2.237 | 1.479 | [34] | [34] |
| CSE Asia | India | Khandera | 28.5230 | 77.6370 | 2004 | 2006 | Fortnightly | N | 3 | 4000 | 1.500 | 0.000 | 0.007 | 1.802 | [34] | [34] |
| CSE Asia | India | Mubarakpur (control) | 28.6830 | 77.5320 | 1999 | 2000 | Fortnightly | N | 28 | 1020 | 54.902 |  | 7.399 | 7.399 | [35] | [3] |
| CSE Asia | India | Shiddhipur (UTnets) | 28.6500 | 77.6500 | 1999 | 2000 | Fortnightly | N | 14 | 1950 | 14.359 |  | 10.787 | 10.787 | [35] | [3] |
| CSE Asia | India | Piyawali (Control) | 28.5490 | 77.5540 | 1999 | 2000 | Weekly | N | 82 | 6520 | 12.577 |  | 7.162 | 7.162 | [36] | [3] |
| CSE Asia | India | Jadoanpur (Control) | 28.6500 | 77.6500 | 1995 | 1996 | Weekly | N | 19 | 693 | 27.417 |  | 19.412 | 19.412 | [37] | [3] |
| CSE Asia | India | Sundargarh (plains) | 22.2474 | 84.7465 | 2001 | 2003 | Weekly | Y | 210 | 6489 | 32.362 | 1.262 | 1.654 | 1.765 | [38] | [38] |
| CSE Asia | India | Sundargarh (forest) | 22.0073 | 84.8111 | 2001 | 2003 | Weekly | Y | 1828 | 6174 | 296.080 | 10.670 | 13.986 | 12.408 | [38] | [38] |
| CSE Asia | India | Sundargarh (plains II) | 22.2389 | 84.7428 | 2001 | 2001 | Weekly | Y | 86 | 2163 | 39.760 | 1.899 | 2.489 | 2.439 | [39] | [39] |
| CSE Asia | India | Sundargarh (forest II) | 22.0076 | 84.8150 | 2001 | 2001 | Weekly | Y | 611 | 2058 | 296.890 | 10.849 | 14.220 | 15.043 | [39] | [39] |
| CSE Asia | India | Banganatham | 13.1040 | 77.9820 | 1994 | 1994 | Fortnightly | Y | 1 | 390 | 2.564 |  | 11.198 | 11.198 | [40] | [3] |
| CSE Asia | India | Bodapatti | 13.1410 | 78.1360 | 1994 | 1996 | Fortnightly | Y | 3 | 1188 | 2.525 |  | 8.881 | 8.881 | [40] | [3] |
| CSE Asia | India | Puram | 13.1480 | 78.2960 | 1994 | 1996 | Fortnightly | Y | 15 | 1194 | 12.563 |  | 11.251 | 11.251 | [40] | [3] |
| CSE Asia | India | Jeypore (Riverine) | 19.1811 | 82.5268 | 1988 | 1989 | Fortnightly | N | 114 | 2441 | 93.404 | 2.162 | 2.834 | 32.222 | [41] | [42] |
| CSE Asia | India | Jeypore (Top hill) | 18.9827 | 82.7162 | 1988 | 1989 | Fortnightly | N | 64 | 1234 | 103.728 | 2.556 | 3.351 | 32.181 | [41] | [42] |
| CSE Asia | India | Jeypore (Foothill) | 19.7854 | 82.1661 | 1988 | 1989 | Fortnightly | N | 449 | 2360 | 380.508 | 5.908 | 7.744 | 31.197 | [41] | [42] |
| CSE Asia | India | Malkangiri (Plain) | 18.6039 | 82.0804 | 1988 | 1989 | Fortnightly | N | 97 | 712 | 272.472 |  | 33.651 | 33.651 | [41] | [3] |
| CSE Asia | India | Malkingiri (foot hill) | 18.2793 | 82.3059 | 1988 | 1989 | Fortnightly | N | 152 | 377 | 806.366 | 33.923 | 44.465 | 34.048 | [41] | [42] |
| CSE Asia | India | Malkingiri (riverine) | 17.8193 | 81.5799 | 1988 | 1989 | Fortnightly | N | 91 | 1137 | 160.070 | 37.885 | 49.658 | 31.380 | [41] | [42] |
| CSE Asia | India | Malkangiri (tophill) | 18.2748 | 82.1977 | 1988 | 1989 | Fortnightly | N | 50 | 118 | 847.458 | 44.554 | 58.400 | 34.379 | [41] | [42] |
| CSE Asia | India | Jeypore (tophill) | 18.9827 | 82.7162 | 1988 | 1989 | Fortnightly | N | 285 | 698 | 816.619 | 49.096 | 64.352 | 32.275 | [41] | [42] |
| CSE Asia | India | Jeypore | 18.9827 | 82.7162 | 1987 | 1987 | Fortnightly | N | 1080 | 15303 | 141.149 | 4.812 | 6.307 | 33.186 | [42] | [42] |
| CSE Asia | India | Birkera | 22.1330 | 84.7990 | 2003 | 2003 | Fortnightly | N | 42 | 367 | 228.883 | 17.166 | 22.501 | 4.792 | [43] | [43] |
| CSE Asia | India | Dudurta | 22.2431 | 85.0447 | 2003 | 2003 | Fortnightly | N | 33 | 506 | 130.435 | 3.162 | 4.145 | 10.606 | [43] | [43] |
| CSE Asia | India | San Pokhari | 22.4583 | 85.1911 | 2003 | 2003 | Fortnightly | N | 29 | 271 | 214.022 | 10.886 | 14.268 | 9.988 | [43] | [43] |
| CSE Asia | India | Kuaramunda (area 1 Control) | 22.3010 | 84.7830 | 1989 | 1990 | Weekly | N | 125 | 626 | 199.681 | 27.273 | 35.748 | 13.710 | [44] | [44] |
| CSE Asia | India | Kuaramunda (area 1 Control) | 22.3010 | 84.7830 | 1990 | 1991 | Weekly | N | 286 | 786 | 363.868 | 36.893 | 48.358 | 12.523 | [44] | [44] |
| CSE Asia | India | Kuaramunda (area 1 Control) | 22.3010 | 84.7830 | 1991 | 1992 | Weekly | N | 231 | 808 | 285.891 | 14.729 | 19.306 | 11.663 | [44] | [44] |
| CSE Asia | India | Kuaramunda (area 1 Control) | 22.3010 | 84.7830 | 1992 | 1993 | Weekly | N | 155 | 814 | 190.418 | 14.729 | 19.306 | 11.199 | [44] | [44] |
| CSE Asia | India | Kuaramunda (area 2 untreated) | 22.3010 | 84.7830 | 1989 | 1990 | Weekly | N | 250 | 1089 | 229.568 | 17.021 | 22.311 | 13.686 | [44] | [44] |
| CSE Asia | India | Kuaramunda (area 2 untreated) | 22.3010 | 84.7830 | 1990 | 1991 | Weekly | N | 285 | 1226 | 232.463 | 15.217 | 19.946 | 12.545 | [44] | [44] |
| CSE Asia | India | Kuaramunda (area 2 untreated) | 22.3010 | 84.7830 | 1991 | 1992 | Weekly | N | 211 | 1328 | 158.886 | 9.865 | 12.931 | 11.646 | [44] | [44] |
| CSE Asia | India | Kuaramunda (area 2 untreated) | 22.3010 | 84.7830 | 1992 | 1993 | Weekly | N | 118 | 1403 | 84.105 | 9.865 | 12.931 | 11.216 | [44] | [44] |
| CSE Asia | India | Kuaramunda (area 3 treated ) | 22.3010 | 84.7830 | 1989 | 1990 | Weekly | N | 289 | 1134 | 254.850 | 19.435 | 25.474 | 13.746 | [44] | [44] |
| CSE Asia | India | Kuaramunda (area 3 treated ) | 22.3010 | 84.7830 | 1990 | 1991 | Weekly | N | 156 | 1147 | 136.007 | 10.920 | 14.313 | 12.542 | [44] | [44] |
| CSE Asia | India | Kuaramunda (area 3 treated ) | 22.3010 | 84.7830 | 1991 | 1992 | Weekly | N | 156 | 1187 | 131.424 | 7.285 | 9.548 | 11.634 | [44] | [44] |
| CSE Asia | India | Kuaramunda (area 3 treated ) | 22.3010 | 84.7830 | 1992 | 1993 | Weekly | N | 75 | 1220 | 61.475 | 7.285 | 9.548 | 11.205 | [44] | [44] |
| CSE Asia | India | Janghira | 21.4333 | 85.6166 | 1994 | 1994 | Fortnightly | Y | 1279 | 28225 | 45.314 | 8.389 | 10.996 | 17.878 | [45] | [45] |
| CSE Asia | India | Jiranga | 18.9827 | 82.7162 | 1994 | 1996 | Fortnightly | Y | 2351 | 67566 | 34.796 | 8.925 | 11.699 | 25.490 | [45] | [45] |
| CSE Asia | India | B.Singpur | 21.6830 | 85.2650 | 1987 | 1988 | Fortnightly | N | 252 | 1476 | 341.463 | 10.601 | 13.895 | 36.642 | [46] | [47] |
| CSE Asia | India | Sonapur | 26.1160 | 91.9820 | 1990 | 1992 | Weekly | N | 8026 | 67545 | 118.824 | 13.972 | 18.314 | 31.871 | [48] | [49] |
| CSE Asia | India | Medeluanjan and Paninara | 26.6010 | 94.1990 | 2001 | 2002 | Weekly | Y | 174 | 2625 | 66.286 |  | 18.008 | 18.008 | [50] | [3] |
| CSE Asia | India | San Dulakudar | 22.1749 | 84.7850 | 1998 | 1999 | Weekly | Y | 197 | 264 | 746.212 | 19.118 | 25.058 | 11.166 | [51] | [51] |
| CSE Asia | India | San Dulakudar | 22.1749 | 84.7850 | 1999 | 2000 | Weekly | Y | 156 | 263 | 593.156 | 20.833 | 27.307 | 9.583 | [51] | [51] |
| CSE Asia | India | San Dulakudar | 22.1749 | 84.7850 | 2000 | 2001 | Weekly | Y | 159 | 266 | 597.744 | 13.443 | 17.620 | 7.719 | [39] | [51] |
| CSE Asia | India | San Dulakudar | 22.1749 | 84.7850 | 2001 | 2002 | Weekly | Y | 174 | 271 | 643.900 | 17.606 | 23.076 | 6.003 | [52] |  |
| CSE Asia | India | San Dulakudar | 22.1749 | 84.7850 | 2002 | 2003 | Weekly | Y | 105 | 271 | 387.454 | 4.790 | 6.279 | 4.266 | [52] |  |
| CSE Asia | India | San Dulakudar | 22.1749 | 84.7850 | 2003 | 2004 | Weekly | Y | 70 | 271 | 258.303 | 5.014 | 6.572 | 2.562 | [52] |  |
| CSE Asia | India | San Dulakudar | 22.1749 | 84.7850 | 2004 | 2005 | Weekly | Y | 47 | 271 | 173.432 | 3.860 | 5.059 | 2.212 | [52] |  |
| CSE Asia | India | San Dulakudar | 22.1749 | 84.7850 | 2005 | 2006 | Weekly | Y | 64 | 271 | 235.000 | 2.899 | 3.799 | 2.992 | [52] |  |
| CSE Asia | India | Pandaripathar and Jagda (control) | 21.9932 | 84.7985 | 2001 | 2002 | Weekly | Y | 82 | 299 | 274.800 | 18.862 | 24.724 | 14.430 | [52] |  |
| CSE Asia | India | Pandaripathar and Jagda (control) | 21.9932 | 84.7985 | 2002 | 2003 | Weekly | Y | 92 | 299 | 307.692 | 19.817 | 25.975 | 11.494 | [52] |  |
| CSE Asia | India | Pandaripathar and Jagda (control) | 21.9932 | 84.7985 | 2003 | 2004 | Weekly | Y | 93 | 299 | 311.037 | 15.831 | 20.751 | 8.326 | [52] |  |
| CSE Asia | India | Pandaripathar and Jagda (control) | 21.9932 | 84.7985 | 2004 | 2005 | Weekly | Y | 98 | 299 | 327.759 | 19.874 | 26.049 | 7.514 | [52] |  |
| CSE Asia | India | Pandaripathar and Jagda (control) | 21.9932 | 84.7985 | 2005 | 2006 | Weekly | Y | 81 | 299 | 270.903 | 14.377 | 18.845 | 8.518 | [52] |  |
| CSE Asia | India | Duduwa | 22.9593 | 80.1923 | 1987 | 1995 | Weekly | Y | 412 | 4671 | 88.204 |  | 18.856 | 18.856 | [53] | [3] |
| CSE Asia | India | Khapa | 22.9908 | 80.1457 | 1987 | 1995 | Weekly | Y | 394 | 2745 | 143.534 |  | 18.712 | 18.712 | [53] | [3] |
| CSE Asia | India | Magardha | 22.9839 | 80.1912 | 1987 | 1995 | Weekly | Y | 457 | 7470 | 61.178 |  | 18.750 | 18.750 | [53] | [3] |
| CSE Asia | India | Tarwani | 22.9660 | 80.1399 | 1987 | 1995 | Weekly | Y | 288 | 3870 | 74.419 |  | 18.796 | 18.796 | [53] | [3] |
| CSE Asia | India | Bisra | 22.2660 | 84.9830 | 1988 | 1989 | Weekly | ? | 5905 | 41282 | 143.041 | 23.784 | 23.784 | 29.540 | [54] | [54] |
| CSE Asia | India | Malkangiri (area 1 un) | 18.3800 | 82.1860 | 1998 | 1999 | Fortnightly | N | 107 | 495 | 432.323 | 35.948 | 37.028 | 24.471 | [55] | [55] |
| CSE Asia | India | Malkangiri (area 1 un) | 18.3800 | 82.1860 | 1999 | 2000 | Fortnightly | N | 94 | 454 | 414.325 | 44.586 | 45.925 | 23.869 | [55] | [55] |
| CSE Asia | India | Malkangiri (area 2 tn) | 18.3800 | 82.1860 | 1998 | 1999 | Fortnightly | N | 61 | 489 | 249.489 | 44.000 | 45.322 | 24.371 | [55] | [55] |
| CSE Asia | India | Malkangiri (area 2 tn) | 18.3800 | 82.1860 | 1999 | 2000 | Fortnightly | N | 21 | 448 | 93.698 | 17.460 | 17.985 | 23.900 | [55] | [55] |
| CSE Asia | India | Malkangiri (control nn) | 18.3800 | 82.1860 | 1998 | 1999 | Fortnightly | N | 51 | 501 | 203.593 | 42.609 | 43.889 | 24.466 | [55] | [55] |
| CSE Asia | India | Malkangiri (control nn) | 18.3800 | 82.1860 | 1999 | 2000 | Fortnightly | N | 69 | 459 | 300.490 | 60.976 | 62.807 | 23.911 | [55] | [55] |
| CSE Asia | India | Malkangiri (area 1 un) | 18.3800 | 82.1860 | 2000 | 2001 | Fortnightly | N | 94 | 528 | 356.061 | 52.632 | 55.766 | 23.298 | [56] | [56] |
| CSE Asia | India | Malkangiri (area 1 un) | 18.3800 | 82.1860 | 2001 | 2003 | Fortnightly | N | 213 | 924 | 461.039 | 33.094 | 35.064 | 21.486 | [56] | [56] |
| CSE Asia | India | Malkangiri (area 2 tn) | 18.3800 | 82.1860 | 2000 | 2001 | Fortnightly | N | 90 | 497 | 362.173 | 40.000 | 42.382 | 23.319 | [56] | [56] |
| CSE Asia | India | Malkangiri (area 2 tn) | 18.3800 | 82.1860 | 2001 | 2003 | Fortnightly | N | 61 | 870 | 140.270 | 15.719 | 16.655 | 21.506 | [56] | [56] |
| CSE Asia | India | Malkangiri (control nn) | 18.3800 | 82.1860 | 2000 | 2001 | Fortnightly | N | 69 | 590 | 233.898 | 32.468 | 34.401 | 23.266 | [56] | [56] |
| CSE Asia | India | Malkangiri (control nn) | 18.3800 | 82.1860 | 2001 | 2003 | Fortnightly | N | 356 | 1033 | 689.588 | 53.145 | 56.309 | 21.541 | [56] | [56] |
| CSE Asia | India | Kurseong | 26.8850 | 88.2780 | 2000 | 2001 | Fortnightly | Y | 101 | 82117 | 1.230 |  | 0.124 | 0.124 | [57] | [3] |
| CSE Asia | India | Kurseong | 26.8850 | 88.2780 | 2001 | 2002 | Fortnightly | Y | 175 | 83797 | 2.088 |  | 0.092 | 0.092 | [57] | [3] |
| CSE Asia | India | Kurseong | 26.8850 | 88.2780 | 2002 | 2003 | Fortnightly | Y | 219 | 85504 | 2.561 |  | 0.066 | 0.066 | [57] | [3] |
| CSE Asia | India | Kurseong | 26.8850 | 88.2780 | 2003 | 2004 | Fortnightly | Y | 590 | 87248 | 6.762 |  | 0.042 | 0.042 | [57] | [3] |
| CSE Asia | India | Kurseong | 26.8850 | 88.2780 | 2004 | 2005 | Fortnightly | Y | 539 | 89028 | 6.054 |  | 0.025 | 0.025 | [57] | [3] |
| CSE Asia | Iran | Ghassareghand | 26.0708 | 60.7917 | 1995 | 1995 | Every 10 days | Y | 13 | 2900 | 4.483 |  | 9.002 | 9.002 | [58] | [3] |
| CSE Asia | Myanmar | Oo-Do | 17.1140 | 96.2550 | 1995 | 1997 | Weekly | N | 91 | 348 | 261.494 | 36.421 | 47.738 | 38.062 | [59] | [59] |
| CSE Asia | Sri Lanka | Mahameegaswewa | 8.1336 | 80.7459 | 1994 | 1995 | Every 2nd day | Y | 122 | 280 | 124.490 | 4.643 | 6.086 | 5.278 | [60] | [61] |
| CSE Asia | Thailand | Mae-Ramand District | 16.9167 | 98.6667 | 1993 | 1994 | Weekly | Y | 71 | 757 | 93.791 |  | 13.763 | 13.763 | [62] | [3] |
| CSE Asia | Thailand | Shoklo refugee camp | 17.4160 | 98.1760 | 1991 | 1992 | Weekly | Y | 121 | 356 | 339.888 | 8.411 | 11.025 | 14.003 | [63] | [63] |
| CSE Asia | Thailand | Shoklo refugee camp | 17.4160 | 98.1760 | 1992 | 1992 | Weekly | Y | 130 | 345 | 376.812 | 6.590 | 6.983 | 14.357 | [64] | [65] |
| CSE Asia | Thailand | Bo Thong | 13.2670 | 101.4320 | 1989 | 1989 | Weekly | Y | 33 | 242 | 136.364 | 9.486 | 12.434 | 10.509 | [66] | [66] |
| CSE Asia | Thailand | Bo Thong | 13.2670 | 101.4320 | 1989 | 1990 | Weekly | Y | 8 | 242 | 33.058 | 9.486 | 12.434 | 10.207 | [66] | [66] |
| CSE Asia | Thailand | Bo Thong | 13.2670 | 101.4320 | 1987 | 1988 | Weekly | N | 12 | 82 | 145.985 | 9.486 | 12.434 | 11.305 | [67] | [66] |
| CSE Asia | Thailand | Pong Nam Ron (Klong takon and Na Jork) | 12.9160 | 102.3820 | 1989 | 1990 | Weekly | Y | 21 | 421 | 49.881 | 11.086 | 14.532 | 9.970 | [68] | [68] |
| CSE Asia | Vietnam | Phu Cuong | 15.1431 | 108.1367 | 1999 | 2000 | Weekly | Y | 0 | 750 | 0.000 |  | 2.788 | 2.788 | [69] | [3] |
| CSE Asia | Vietnam | An Trach | 9.1838 | 105.3194 | 1999 | 2001 | Weekly | Y | 12 | 910 | 13.184 | 0.415 | 0.544 | 0.516 | [70] | [70] |
| CSE Asia | Vietnam | Suoi Kiet | 10.9670 | 107.6330 | 1999 | 2001 | Weekly | Y | 102 | 957 | 106.616 | 1.424 | 1.867 | 2.081 | [71] | [71] |

**Footnotes:**

Region Global region of active case detection (ACD) study site [3]

Country Country of ACD study site

ACD location Place name of ACD study site

Latitude Latitude of ACD study site

Longitude Longitude of ACD study site

Start year Start year of ACD study

End year End year of ACD study

Freq of ACD Frequency of ACD

PCD Passive case detection

*d* Number of cases

PYO Person years of observation

*γ* Incidence per 1000 per annum, scaled for the frequency of surveillance

*Pf*PR *Plasmodium falciparum* parasite rate recorded

*Pf*PR_2-10_ *Plasmodium falciparum* parasite rate standardized to the two up to ten age-range

*Pf*PR_2-10_ pred *Plasmodium falciparum* parasite rate standardized to the two up to ten age-range predicted using methods outlined in Hay *et al*. [3]

Ref ACD Reference for the active case detection study

Ref *Pf*PR Reference for the *Plasmodium falciparum* parasite rate

* Studies where the details of surveillance were unclear and assumed from other sources of information; or studies with aggressive, focused health service provision to small defined communities equivalent to seeing all population weekly

**References**

Titles in square brackets have been translated into English.

1. Ernst KC, Adoka SO, Kowuor DO, Wilson ML, John CC: **Malaria hotspot areas in a highland Kenya site are consistent in epidemic and non-epidemic years and are associated with ecological factors**. *Malar J* 2006, **5**:78.

2. John CC: **Personal communication**. Minneapolis, U.S.A.: Department of Paediatrics, University of Minnesota; 2008.

3. Hay SI, Guerra CA, Gething PW, Patil AP, Tatem AJ, Noor AM, Kabaria CW, Manh BH, Elyazar IRF, Brooker SJ, Smith DL, Moyeed RA, Snow RW: **A world malaria map: *Plasmodium falciparum* endemicity in 2007**. *PLoS Med* 2009, **6**:e1000048.

4. Macintyre K, Sosler S, Letipila F, Lochigan M, Hassig S, Omar SA, Githure J: **A new tool for malaria prevention?: Results of a trial of permethrin-impregnated bedsheets (*shukas*) in an area of unstable transmission**. *Int J Epidemiol* 2003, **32**:157-160.

5. Mwangi TW: **Clinical epidemiology of malaria under differing levels of transmission**. Oxford, U.K.: D.Phil. thesis, Weatherall Institute of Molecular Medicine, University of Oxford; 2003.

6. Mwangi TW, Mohammed M, Dayo H, Snow RW, Marsh K: **Clinical algorithms for malaria diagnosis lack utility among people of different age groups**. *Trop Med Int Health* 2005, **10**:530-536.

7. Mwangi TW, Ross A, Snow RW, Marsh K: **Case definitions of clinical malaria under different transmission conditions in Kilifi District, Kenya**. *J Infect Dis* 2005, **191**:1932-1939.

8. Boisier P, Jambou R, Raharimalala L, Roux J: **Relationship between parasite density and fever risk in a community exposed to a low level of malaria transmission in Madagascar highlands**. *Am J Trop Med Hyg* 2002, **67**:137-140.

9. Dicko A, Sagara I, Diemert D, Sogoba M, Niambele MB, Dao A, Dolo G, Yalcouye D, Diallo DA, Saul A, Miller LH, Toure YT, Klion AD, Doumbo OK: **Year-to-year variation in the age-specific incidence of clinical malaria in two potential vaccine testing sites in Mali with different levels of malaria transmission intensity**. *Am J Trop Med Hyg* 2007, **77**:1028-1033.

10. Dolo A, Modiano D, Maiga B, Daou M, Dolo G, Guindo H, Ba M, Maiga H, Coulibaly D, Perlman H, Blomberg MT, Toure YT, Coluzzi M, Doumbo O: **Difference in susceptibility to malaria between two sympatric ethnic groups in Mali**. *Am J Trop Med Hyg* 2005, **72**:243-248.

11. Coulibaly D, Diallo DA, Thera MA, Dicko A, Guindo AB, Kone AK, Cissoko Y, Coulibaly S, Djimde A, Lyke K, Doumbo OK, Plowe CV: **Impact of preseason treatment on incidence of falciparum malaria and parasite density at a site for testing malaria vaccines in Bandiagara, Mali**. *Am J Trop Med Hyg* 2002, **67**:604-610.

12. Dicko AA: **[Malaria epidemiology in Mopti region with a view to developing a regional control programme]**. Bamako, Mali: Ph.D. thesis, Ecole Nationale de Médecine et de Pharmacie du Mali (ENMP); 1995.

13. Diallo S, Konate L, Ndir O, Dieng T, Dieng Y, Bah IB, Faye O, Gaye O: **[Malaria in the central health district of Dakar (Senegal). Entomological, parasitological and clinical data]**. *Cahiers Santé* 2000, **10**:221-229.

14. Ba F: **[Malaria in a mesoendemic region: relationship between disease transmission, infection and morbidity in Ndiop (Senegal)]**. Dakar, Senegal: Ph.D. thesis, Falculté des Sciences et Techniques, Université Cheikh Anta Diop de Dakar; 2000.

15. Molez JF, Diop A, Gaye O, Lemasson JJ, Fontenille D: **[Malaria morbidity in Barkedji, village of Ferlo, in Senegal Sahelian area]**. *Bulletin de la Société de Pathologie Exotique* 2006, **99**:187-190.

16. Mnzava AE, Sharp BL, Mthembu DJ, le Sueur D, Dlamini SS, Gumede JK, Kleinschmidt I: **Malaria control--two years' use of insecticide-treated bednets compared with insecticide house spraying in KwaZulu-Natal**. *S Afr Med J* 2001, **91**:978-983.

17. Giha HA, Rosthoj S, Dodoo D, Hviid L, Satti GM, Scheike T, Arnot DE, Theander TG: **The epidemiology of febrile malaria episodes in an area of unstable and seasonal transmission**. *Trans R Soc Trop Med Hyg* 2000, **94**:645-651.

18. Salcedo JM, Camargo EP, Krieger H, Silva LH, Camargo LM: **Malaria control in an agro-industrial settlement of Rondonia (Western Amazon region, Brazil)**. *Mem Inst Oswaldo Cruz* 2000, **95**:139-145.

19. Camargo LM, Ferreira MU, Krieger H, De Camargo EP, Da Silva LP: **Unstable hypoendemic malaria in Rondonia (western Amazon region, Brazil): epidemic outbreaks and work-associated incidence in an agro-industrial rural settlement**. *Am J Trop Med Hyg* 1994, **51**:16-25.

20. da Silva-Nunes M, Ferreira MU: **Clinical spectrum of uncomplicated malaria in semi-immune Amazonians: beyond the "symptomatic" vs "asymptomatic" dichotomy**. *Mem Inst Oswaldo Cruz* 2007, **102**:341-347.

21. da Silva-Nunes M, S. MR, A. LB, Souza EA, Martins LC, Rodrigues SG, Chiang JO, Vasconcelos PF, Muniz PT, Ferreira MU: **The Acre Project: the epidemiology of malaria and arthropod-borne virus infections in a rural Amazonian population**. *Cad Saude Publica* 2006, **22**:1325-1334.

22. Cardoso MA, Ferreira MU, Camargo LM, Szarfarc SC: **Anaemia, iron deficiency and malaria in a rural community in Brazilian Amazon**. *Eur J Clin Nutr* 1994, **48**:326-332.

23. Alves FP, Durlacher RR, Menezes MJ, Krieger H, Silva LH, Camargo EP: **High prevalence of asymptomatic *Plasmodium vivax* and *Plasmodium falciparum* infections in native Amazonian populations**. *Am J Trop Med Hyg* 2002, **66**:641-468.

24. Duarte EC, Gyorkos TW, Pang L, Abrahamowicz M: **Epidemiology of malaria in a hypoendemic Brazilian Amazon migrant population: a cohort study**. *Am J Trop Med Hyg* 2004, **70**:229-237.

25. Tada MS, Marques RP, Mesquita E, Dalla Martha RC, Rodrigues JA, Costa JD, Pepelascov RR, Katsuragawa TH, Pereira-da-Silva LH: **Urban malaria in the Brazilian Western Amazon Region I: high prevalence of asymptomatic carriers in an urban riverside district is associated with a high level of clinical malaria**. *Mem Inst Oswaldo Cruz* 2007, **102**:263-369.

26. Richards FO, Jr., Klein RE, Flores RZ, Weller S, Gatica M, Zeissig R, Sexton J: **Permethrin-impregnated bed nets for malaria control in northern Guatemala: epidemiologic impact and community acceptance**. *Am J Trop Med Hyg* 1993, **49**:410-418.

27. Guthmann JP, Llanos-Cuentas A, Palacios A, Hall AJ: **Environmental factors as determinants of malaria risk. A descriptive study on the northern coast of Peru**. *Trop Med Int Health* 2002, **7**:518-525.

28. Roper MH, Torres RS, Goicochea CG, Andersen EM, Guarda JS, Calampa C, Hightower AW, Magill AJ: **The epidemiology of malaria in an epidemic area of the Peruvian Amazon**. *Am J Trop Med Hyg* 2000, **62**:247-256.

29. Noya O, Gabaldon Berti Y, Alarcon de Noya B, Borges R, Zerpa N, Urbaez JD, Madonna A, Garrido E, Jimenez MA, Borges RE, et al.: **A population-based clinical trial with the SPf66 synthetic *Plasmodium falciparum* malaria vaccine in Venezuela**. *J Infect Dis* 1994, **170**:396-402.

30. Camargo LM, dal Colletto GM, Ferreira MU, Gurgel Sde M, Escobar AL, Marques A, Krieger H, Camargo EP, da Silva LH: **Hypoendemic malaria in Rondonia (Brazil, western Amazon region): seasonal variation and risk groups in an urban locality**. *Am J Trop Med Hyg* 1996, **55**:32-38.

31. Magris M, Rubio-Palis Y, Alexander N, Ruiz B, Galvan N, Frias D, Blanco M, Lines J: **Community-randomized trial of lambdacyhalothrin-treated hammock nets for malaria control in Yanomami communities in the Amazon region of Venezuela**. *Trop Med Int Health* 2007, **12**:392-403.

32. Laserson KF, Wypij D, Petralanda I, Spielman A, Maguire JH: **Differential perpetuation of malaria species among Amazonian Yanomami Amerindians**. *Am J Trop Med Hyg* 1999, **60**:767-773.

33. Neng W, Liexin Q, Guohou L, Weimin Z, Wenkui G, Yuming S, Yi T, Kuanren Z: **Field evaluation of bednets impregnated with deltamethrin for malaria control**. *Southeast Asian J Trop Med Public Health* 1993, **24**:664-671.

34. Sreehari U, Razdan RK, Mittal PK, Ansari MA, Rizvi MM, Dash AP: **Impact of Olyset Nets on malaria transmission in India**. *J Vector Borne Dis* 2007, **44**:137-144.

35. Ansari MA, Razdan RK: **Bio-efficacy and operational feasibility of alphacypermethrin (Fendona) impregnated mosquito nets to control rural malaria in northern India**. *J Vector Borne Dis* 2003, **40**:33-42.

36. Ansari MA, Razdan RK: **Impact of residual spraying of Reldan against *Anopheles culicifacies* in selected villages of District Ghaziabad (Uttar Pradesh), India**. *J Vector Borne Dis* 2004, **41**:54-60.

37. Ansari MA, Razdan RK: **Operational feasibility and efficacy of Deltamethrin impregnated hessian curtains in comparison to HCH indoor residual spraying to control malaria in selected villages of District Ghaziabad (U.P.), India**. *Indian J Malariol* 2000, **37**:1-10.

38. Sharma SK, Tyagi PK, Padhan K, Upadhyay AK, Haque MA, Nanda N, Joshi H, Biswas S, Adak T, Das BS, Chauhan VS, Chitnis CE, Subbarao SK: **Epidemiology of malaria transmission in forest and plain ecotype villages in Sundargarh District, Orissa, India**. *Trans R Soc Trop Med Hyg* 2006, **100**:917-925.

39. Sharma SK, Tyagi PK, Padhan K, Adak T, Subbarao SK: **Malarial morbidity in tribal communities living in the forest and plain ecotypes of Orissa, India**. *Ann Trop Med Parasitol* 2004, **98**:459-468.

40. Ghosh SK, Tiwari SN, Sathyanarayan TS, Sampath TR, Sharma VP, Nanda N, Joshi H, Adak T, Subbarao SK: **Larvivorous fish in wells target the malaria vector sibling species of the *Anopheles culicifacies* complex in villages in Karnataka, India**. *Trans R Soc Trop Med Hyg* 2005, **99**:101-115.

41. Jambulingam P, Mohapatra SSS, Govardhini P, Das LK, Manoharan A, Pani SP, Das PK: **Microlevel epidemiological variations in malaria & its implications on control strategy**. *Indian J Med Res* 1991, **93**:371-378.

42. Pani SP: **Epidemiology of malaria persistence in Koraput District, Orissa State**. Pondicherry, India: Pondicherry University 1990.

43. Sharma SK, Upadhyay AK, Haque MA, Padhan K, Tyagi PK, Batra CP, Adak T, Dash AP, Subbarao SK: **Effectiveness of mosquito nets treated with a tablet formulation of deltamethrin for malaria control in a hyperendemic tribal area of Sundargarh District, Orissa, India**. *J Am Mosq Control Assoc* 2006, **22**:111-118.

44. Yadav RS, Sampath TR, Sharma VP, Adak T, Ghosh SK: **Evaluation of lambdacyhalothrin-impregnated bednets in a malaria endemic area of India. Part 3. Effects on malaria incidence and clinical measures**. *J Am Mosq Control Assoc* 1998, **14**:444-450.

45. Sharma SK: **Approaches to increasing the use on insecticide treated mosquito nets in Orissa, India. Papers presented at a Seminar on 14 and 15 March 1997 Bhubaneswar, Orissa**. In*.* Edited by Rath AD. Bhubaneswar, Orissa, India; 1997.

46. Subramanian S, Manoharan A, Sahu S, Jambulingam P, Govardhini P, Mohapatra SSS, Das PK: **Living conditions and occurrence of malaria in a rural community**. *Indian J Malariol* 1991, **28**:29-37.

47. Rajagopalan PK, Pani SP, Das PK, Jambulingam P: **Malaria in Koraput District of Orissa**. *Indian J Pediatr* 1989, **56**:355-364.

48. Dev V, Phookan S, Sharma VP, Anand SP: **Physiographic and entomologic risk factors of malaria in Assam, India**. *Am J Trop Med Hyg* 2004, **71**:451-456.

49. Dev V, Phookan S, Sharma VP, Dash AP, Anand SP: **Malaria parasite burden and treatment seeking behavior in ethnic communities of Assam, Northeastern India**. *J Infect* 2006, **52**:131-139.

50. Prakash A, Bhattacharyya DR, Mohapatra PK, Mahanta J: **Role of the prevalent *Anopheles* species in the transmission of *Plasmodium falciparum* and *P. vivax* in Assam state, north-eastern India**. *Ann Trop Med Parasitol* 2004, **98**:559-568.

51. Sharma SK, Chattopadhyay R, Chakrabarti K, Pati SS, Srivastava VK, Tyagi PK, Mahanty S, Misra SK, Adak T, Das BS, Chitnis CE: **Epidemiology of malaria transmission and development of natural immunity in a malaria-endemic village, San Dulakudar, in Orissa state, India**. *Am J Trop Med Hyg* 2004, **71**:457-465.

52. Sharma SK, Tyagi PK, Upadhyay AK, Haque MA, Adak T, Dash AP: **Building small dams can decrease malaria: a comparative study from Sundargarh District, Orissa, India**. *Acta Trop* 2008, **107**:174-178.

53. Singh N, Mishra SS, Singh MP, Sharma VP: **Seasonality of *Plasmodium vivax* and *P. falciparum* in tribal villages in central India (1987-1995)**. *Ann Trop Med Parasitol* 2000, **94**:101-112.

54. Yadav RS, Sharma VP, Ghosh SK, Kumar A: **Quartan malaria--an investigation on the incidence of *Plasmodium malariae* in Bisra PHC, District Sundargarh, Orissa**. *Indian J Malariol* 1990, **27**:85-94.

55. Sahu SS, Jambulingam P, Vijayakumar T, Subramanian S, Kalyanasundaram M: **Impact of alphacypermethrin treated bed nets on malaria in villages of Malkangiri district, Orissa, India**. *Acta Trop* 2003, **89**:55-66.

56. Sahu SS, Vijayakumar T, Kalyanasundaram M, Subramanian S, Jambulingam P: **Impact of lambdacyhalothrin capsule suspension treated bed nets on malaria in tribal villages of Malkangiri district, Orissa, India**. *Indian J Med Res* 2008, **128**:262-270.

57. Sharma PK, Ramakrishnan R, Hutin YJ, Gupte MD: **Increasing incidence of malaria in Kurseong, Darjeeling District, West Bengal, India, 2000-2004**. *Trans R Soc Trop Med Hyg* 2008.

58. Zaim M, Ghavami MB, Nazari M, Edrissian GH, Nateghpour M: **Cyfluthrin (EW 050)-impregnated bednets in a malaria control program in Ghassreghand (Baluchistan, Iran)**. *J Am Mosq Control Assoc* 1998, **14**:421-430.

59. Soe-Soe, Khin-Saw-Aye, Htay-Aung, Nay-Win, Tin-Aung, Than-Swe, Roussilhon C, Perignon JL, Druilhe P: **Premunition against *Plasmodium falciparum* in a malaria hyperendemic village in Myanmar**. *Trans R Soc Trop Med Hyg* 2001, **95**:81-84.

60. van der Hoek W, Konradsen F, Dijkstra DS, Amerasinghe PH, Amerasinghe FP: **Risk factors for malaria: a microepidemiological study in a village in Sri Lanka**. *Trans R Soc Trop Med Hyg* 1998, **92**:265-269.

61. Amerasinghe PH, Amerasinghe FP, Konradsen F, Fonseka KT, Wirtz RA: **Malaria vectors in a traditional dry zone village in Sri Lanka**. *Am J Trop Med Hyg* 1999, **60**:421-429.

62. Kamolratanakul P, Butraporn P, Prasittisuk M, Prasittisuk C, Indaratna K: **Cost-effectiveness and sustainability of lambdacyhalothrin-treated mosquito nets in comparison to DDT spraying for malaria control in western Thailand**. *Am J Trop Med Hyg* 2001, **65**:279-284.

63. Luxemburger C, Thwai KL, White NJ, Webster HK, Kyle DE, Maelankirri L, Chongsuphajaisiddhi T, Nosten F: **The epidemiology of malaria in a Karen population on the western border of Thailand**. *Trans R Soc Trop Med Hyg* 1996, **90**:105-111.

64. Nosten F, van Vugt M, Price R, Luxemburger C, Thway KL, Brockman A, McGready R, ter Kuile F, Looareesuwan S, White NJ: **Effects of artesunate-mefloquine combination on incidence of *Plasmodium falciparum* malaria and mefloquine resistance in western Thailand: a prospective study**. *Lancet* 2000, **356**:297-302.

65. Luxemburger C, Perea WA, Delmas G, Pruja C, Pecoul B, Moren A: **Permethrin-impregnated bed nets for the prevention of malaria in schoolchildren on the Thai-Burmese border**. *Trans R Soc Trop Med Hyg* 1994, **88**:155-159.

66. Kamolratanakul P, Dhanamun B, Lertmaharit S, Seublinwong T, Udomsangpetch R, Chirakalwasorn N, Thaithong S: **Malaria in a rural area of eastern Thailand: baseline epidemiological studies at Bo Thong**. *Southeast Asian J Trop Med Public Health* 1992, **23**:783-787.

67. Kamol-Ratanakul P, Prasittisuk C: **The effectiveness of permethrin-impregnated bed nets against malaria for migrant workers in eastern Thailand**. *Am J Trop Med Hyg* 1992, **47**:305-309.

68. Kamolratanakul P, Dhanamun B, Lertmaharit S, Seublingwong T, Udomsangpetch R, Thaithong S: **Epidemiological studies of malaria at Pong Nam Ron, eastern Thailand**. *The Southeast Asian Journal of Tropical Medicine and Public Health* 1994, **25**:425-429.

69. Erhart A, Thang ND, Xa NX, Thieu NQ, Hung LX, Hung NQ, Nam NV, Toi LV, Tung NM, Bien TH, Tuy TQ, Cong LD, Thuan LK, Coosemans M, D'Alessandro U: **Accuracy of the health information system on malaria surveillance in Vietnam**. *Trans R Soc Trop Med Hyg* 2007, **101**:216-225.

70. Erhart A, Thang ND, Bien TH, Tung NM, Hung NQ, Hung LX, Tuy TQ, Speybroeck N, Cong LD, Coosemans M, D'Alessandro U: **Malaria epidemiology in a rural area of the Mekong Delta: a prospective community-based study**. *Trop Med Int Health* 2004, **9**:1081-1090.

71. Erhart A, Thang ND, Hung NQ, Toi LV, Hung LX, Tuy TQ, Cong le D, Speybroeck N, Coosemans M, D'Alessandro U: **Forest malaria in Vietnam: a challenge for control**. *Am J Trop Med Hyg* 2004, **70**:110-118.
